# The perceived impact of climate change on mental health and suicidality in Kenyan high school students

**DOI:** 10.1186/s12888-024-05568-8

**Published:** 2024-02-12

**Authors:** David M. Ndetei, Danuta Wasserman, Victoria Mutiso, Jenelle R. Shanley, Christine Musyimi, Pascalyne Nyamai, Timothy Munyua, Monica H. Swahn, John R. Weisz, Tom L. Osborn, Kamaldeep Bhui, Natalie E. Johnson, Panu Pihkala, Peter Memiah, Sonja Gilbert, Afzal Javed, Andre Sourander

**Affiliations:** 1 Africa Mental Health Research and Training Foundation, Mawensi Garden, Mawensi Road, Off Elgon Road, P.O. Box, Nairobi, 48423-00100 Kenya; 2 Department of Psychiatry, University of Nairobi, Nairobi, Kenya; 3 World Psychiatric Association Collaborating Centre for Research and Training, Nairobi, Kenya; 4 Karolinska Institute, Stockholm, Sweden; 5 Pacific University, Hillsboro, USA; 6 Department of Health Promotion and Physical Education, Wellstar College of Health and Human Services, Kennesaw State University, Kennesaw, USA; 7 Department of Psychology, Harvard University, Cambridge, MA USA; 8 Shamiri Institute, Nairobi, Kenya; 9 University of Oxford, Oxford, UK; 10 World Psychiatric Association Collaborating Centre, Oxford, UK; 11 Division of Clinical Epidemiology, Department of Clinical Research, University Hospital Basel, Basel, Switzerland; 12 University of Basel, Basel, Switzerland; 13 Faculty of Theology, University of Helsinki, Helsinki, Finland; 14 University of Maryland, Baltimore, USA; 15 Research Centre for Child Psychiatry, Department of Clinical Medicine, Faculty of Medicine, University of Turku, Turku, Finland; 16 INVEST Child Psychiatry, INVEST Research Flagship Center, Department of Clinical Medicine, Faculty of Medicine, University of Turku, Turku, Finland; 17 World Psychiatric Association, Geneva, Switzerland; 18 Department of Child Psychiatry, Turku University Hospital, Turku, Finland

**Keywords:** Climate change, Mental health, Suicidality, Youth, High-school students, Survey, Kenya

## Abstract

**Background:**

Climate change has psychological impacts but most of the attention has been focused on the physical impact. This study was aimed at determining the association of climate change with adolescent mental health and suicidality as reported by Kenyan high school students.

**Methods:**

This was a cross sectional study with a sample size of 2,652. The participants were high school students selected from 10 schools in 3 regions of Kenya. A questionnaire was used to assess climate change experiences, mental health problems, and suicidality of the youth. Data were analyzed descriptively and with logistic regression to determine various associations of the different variables and the predictors of the various scores of SDQ and suicidality at 95% CI.

**Results:**

Significant differences were observed between gender and two of the threats of climate change – worry and being afraid as subjectively experienced by the participants. Females were more worried and afraid of climate change than males. On univariate and multivariate logistic regression, we found that various experiences of climate change were significantly associated with various scores of SDQ and much fewer of the experiences predicted SDQ scores. The same pattern was reflected in suicidality.

**Conclusion:**

Climate change appears to be associated with mental health concerns and suicidality according to Kenyan high school students’ reports with gender differences in some associations.

## Introduction

Concerns have been raised over the impact of climate change on mental health globally with reports indicating that the psychological impacts of any disaster (climate change included) surpass physical impacts by 40 to 1 [1]. The impacts can be either direct or indirect, short term or long term. The direct impact of climate change on mental health can include the trauma inflicted on the people exposed to climate change related events such as floods, hurricanes, droughts, wild fires, and earthquakes [2, 3]. In addition, increased suicides and mental health related mortality and morbidity have been associated with climate change related events [4–7]. Indirect consequences of climate change include economic loss, threats to physical health and community wellbeing, displacement and forced migration [6, 8–10].

A systematic review of 53 studies on the association between hot weather and poor mental health outcomes established that mental health-related mortality increased by ·2% with a 1^0^C temperature rise [6]. Other studies particularly those with young people have found that climate change evoked emotions such as fear, worry, anger, shame, guilt, disgust, hopelessness and overwhelm [11–13]. The consequences of exposure to extreme and prolonged events related to climate can also be delayed resulting in disorders such as Post-Traumatic Stress Disorder (PTSD), anxiety and depression with the potential to pass such maladies to later generations [7, 9, 14, 15].

Children, adolescents, the elderly, homeless, economically challenged, persons with pre-existing mental conditions and residents of low and middle-income countries have been found to be the most vulnerable to climate change related mental health problems [10, 14, 16, 17]. In addition, gender and location (urban or rural) have been found to be associated with climate change impact with girls and individuals from urban residences more likely to worry about climate change [18]. Despite Africa being among the most vulnerable to climate change, there is little scientific contribution from the continent [19] and even less research about climate emotions in various African countries [20]. A cross-sectional study conducted in six African countries (Ghana, Nigeria, Namibia, South Africa, Ethiopia and Kenya) revealed that more than half the population reported experiencing the impact of climate change [21]. Further, the 2022 report of the Intergovernmental Panel on Climate Change (IPCC) listed East Africa among the global hotspots showing high vulnerability to climate hazards [22]. It is to be noted that East Africa (Kenya included) has experienced floods, increased heat and droughts which have affected infrastructure and forced residents to move in search of stability and safety [19, 23]. This movement and displacement leads to loss of livelihood and property and can thus evoke feelings of hopelessness, helplessness and homesickness, often correlated with mental illness.

In the last decade, Kenya has experienced unprecedented drought leading to food shortages, and loss of livelihoods mainly in pastoral communities which saw their livestock wiped out and others around the country saw reports in the media of wild animals dying for lack of pasture [24]. It was during this period that the present study was conducted.

Our conceptual model is that children’s perception of the negative effects of climate change leads to stress and depending on the severity may lead to mental disorders, which may then lead to suicidality with potential other factors contributing to the process [25]. Despite the potential negative impact of climate change on mental health, much of the research on climate change has been focused on the physical impacts. To our knowledge, there is no reported study with African youths that specifies the various threats posed by climate change, how each of these is related to mental health and whether there are associations with suicidality. To fill this gap, we conducted such a study using a well-established mental health measure for stress related difficulties i.e. (the Strength and Difficulties Questionnaire (SDQ)). The overall objective was to study the association between climate change and mental health. The specific aims were:

1. To determine the prevalence of various perceptions and responses to climate change
2. To assess the association between youth-reported impacts of climate change and SDQ total score and subscale scores for emotional symptoms, conduct problems, hyperactivity, peer problems and prosocial behavior
3. To assess the strength of association between youth-reported climate change impact and suicidality

## Methodology

### Study participants and procedure

Kenya has 47 counties, referred to here as regions. The schools in each county are classified into geographical zones. Each zone is then divided into clusters to facilitate equitable supervision and administrative oversight. In each cluster are to be found different levels of schools. National schools, drawing students from across the country are supervised at the zonal level. In Kenya, High school education is typically divided into four forms: Form 1, Form 2, Form 3, and Form 4. This system is similar to what some countries refer to as grades or years in high school, but in Kenya, they are called "forms." The age range for the students vary from 13 to 20 years due to various factors such as when students began their primary education and whether they repeated any grades. Additionally, students who experience delays or interruptions in their education may also fall within this age range while pursuing their high school studies.

Students from ten secondary (high) schools (*n* = 2,652) participated in this cross-sectional study between May and June 2022. The schools were sampled from three regions out of 47 (otherwise referred to as counties) in Kenya that were conveniently selected to reflect the two broad socioeconomic spaces in the country (urban and rural characteristics). While each of the zones contributed a third of the participants, learning institutions were selected in a non-random approach based on willingness to participate in the research. The age structure is approximately as follows: Form 1 (14–15 years); Form 2 (15–16 years); Form 3 (16–17 years); Form 4 (17–18 years), depending on their actual age. The final list of schools represented all four levels of government-funded schools (national-1 school, extra-county- 1 school, county-5 schools, and sub-county-3 schools) and the socioeconomic spaces (urban-4 and rural characteristics-6). The choice to have more rural schools participate in the study compared to urban schools was deliberate as rural schools are the most vulnerable to the effects of climate change. All students in the sampled schools were randomized between groups of 12–15 students, and led by a research assistant through a permuted block technique. The questionnaire was administered on paper and pencil to the students in a classroom situation who gave informed consent/assent depending on their age.

The following instruments were used:

1. Demographic data were assessed using three self-reported questions: (a) ‘‘Gender?’’ (Male/Female/Other); (b) “Age? (in years)”; (c) ‘‘In what form (high school grade) are you?’’
2. On climate change: We used a tool developed by 11 international consultants with expertise in climate change emotions, clinical and environmental psychology, psychotherapy, psychiatry, human rights law, child and adolescent mental health, and young people with lived experience of climate anxiety [11]. The version of the tool used in the current study had two domains: climate-related worry (level of worry about climate change); and climate-related emotions (presence of 4 negative key emotions about climate change). This questionnaire documented self-reports of threats about climate change using five questions, with the first question having six responses in a 6-point scale 0 = ‘Not worried,’ 1 = ‘A little,’ 2 = ‘Moderately,’ 3 = ‘Very worried,’ 4 = ‘Extremely worried,’ and 5 = ‘Prefer not to say’: “I am worried that climate change threatens people and the planet” and remaining four questions having three responses (‘Yes,’ ‘No,’ ‘Prefer not to say’): “Does climate change make you feel anxious?”; “Does climate change make you feel angry?”; “Does climate change make you feel afraid?”; “Does climate change make you feel powerless?”.
3. Measure of suicidality- This documented suicidal thoughts, plans and attempts. Five questions were asked: (1) “Have you thought seriously about committing suicide?” (‘No, I have not,’ ‘Yes, once,’ ‘Yes, more than once’). For this analysis, the response options were dichotomized into ‘‘No’’ and ‘‘Yes’’; (2) “Have you tried committing suicide?” (‘No, I have not,’ ‘Yes, once,’ ‘Yes, more than once’). For this analysis, the response options were dichotomized into ‘‘No’’and ‘‘Yes’’; (3) “If yes in question 1 above, did you think of a possible way to commit suicide?” (yes/no); (4) “If yes in question 3 above, how?” (list the methods); (5) “If yes in question 2 above, what methods did you use?”. This tool simply asks for the presence or absence of different aspects of suicidality. The questions were added by the Kenyan site to the questionnaire adopted through the process of consultation wth all the PIs in the different countries. We borrowed the questions from one of our Kenyan studies [26].
4. The SDQ – This is a 25-item self-report tool that has been validated to measure prosocial behavior and psychopathology of adolescents and used in many studies across the globe and therefore our study will provide data for global comparison [27–29]. The reliability of this tool is generally satisfactory, whether judged by internal consistency, cross-informant correlation, or retest stability [30]. It is comprised of 5 scales with 5 items each: emotional symptoms, conduct problems, hyperactivity, peer problems, and prosocial behavior. Each item is scored on a 3-point scale with 0 = ‘not true’, 1 = ‘somewhat true’, and 2 = ‘certainly true’. Scores are computed by summing relevant items (after recoding reversed items). For each of the 5 scales, the score can range from 0 to 10 if all 5 items are completed (scale scores can be prorated if at least 3 of the 5 items have been completed). A total difficulties score can also be calculated by summing the scores on the emotional symptoms, conduct problems, hyperactivity-inattention, and peer problems subscales. The total score can range from 0 to 40. We used this tool as published by the authors without any modification and purely for research and without any financial gain. The same tool was used in all the other centers in this cross country multicenter collaborative study.

### Data analysis

Data analysis was performed with SPSS version 25 (Armonk, NY: IBM Corp) for Microsoft Windows®. Descriptive summary statistics in the form of frequency, percentage, mean and standard deviation were generated to examine the variables. Chi-square and Fisher’s exact tests were used where appropriate. Univariate and multivariate logistic regression was done to determine: (i) which socio-demographic variables were associated with experiences of climate change (ii) associations between the various climate change experiences and the various scores on SDQ (iii) associations between various climate change experiences and the various aspects (thoughts, plans and attempts) of suicidality and (iv) which climate change experiences predicted the various scores on SDQ and the various aspects of suicidality.

### Ethics

All procedures contributing to this work complied with the ethical standards of the relevant national and institutional committees on human experimentation. Kenyatta University Ethical Review Committee approved this research (IRB number – PKU/2456/E1587). Permission was sought from institutional heads. Informed consent was obtained from students over 18 years and assent from those under 18 years. In addition, consent was obtained from parents/guardians of participants under 18 years.

## Results

### Socio-demographics

The overall response rate was 97.9% (2596 out of 2652).

Table 1 summarizes the socio-demographics of the participants.

**Table 1:** Socio-demographic characteristics of the participants who respondent to the various socio-demographic variables

Variable	Category	n (%)
Gender	Female	862 (33.2)
	Male	1,728 (66.6)
	Other	6 (0.2)
Age (Years)	Mean (SD)	16.13 (1.38)
	Median (IQR)	16.00 (15.00, 17.00)
	Range	13.00, 23.00
Form (High school class level)	1	869 (33.5)
	2	646 (24.9)
	3	729 (28.1)
	4	352 (13.6)
Location of School	Rural	1,627 (61.3)
	Urban	1,025 (38.7)

A total of 2652 students participated in the study, with a mean age of 16.13 (± 1.38), ranging from 13.00—23.00 More than half of the participants were male (66.6%), with the smallest proportion (13.6%) being form 4’s (the final year in high school) and the biggest proportion living in rural areas (61.3%).

### Threats of climate change

**Fig. 1:**
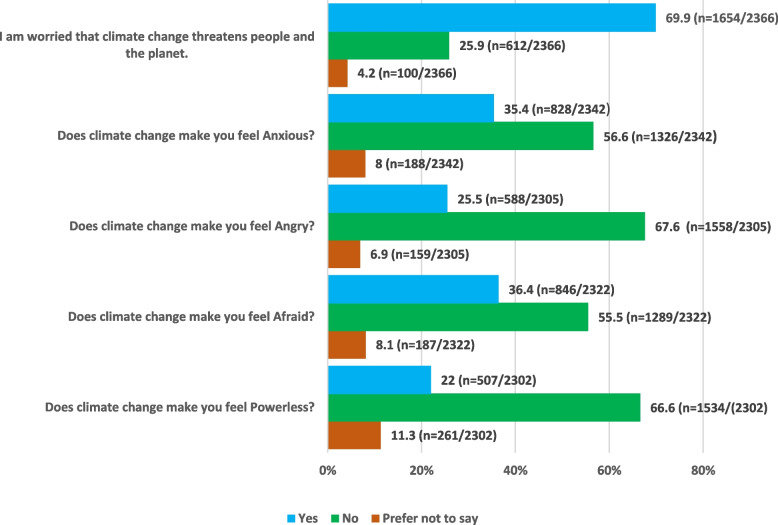
Threats of climate change

Figure 1 summarizes the frequencies of the various and different severity of experiences of climate change.

A majority of the respondents (69.9% (1654/2366)) were worried about climate change. In addition, 35.4% (828/2342), 25.5% (588/2305), 36.4% (846/2322), and 22% (507/2302) of the respondents felt anxious, angry, afraid and powerless respectively in response climate change.

### Climate change stratified by gender and location

There were significant differences by gender in regard to feeling worried and afraid. Males were significantly not worried about climate change compared to females (29% vs. 19.3%). Females were also significantly more afraid of climate change than were males (42.3% vs. 33.8%).

There was a significant difference in worry about climate change depending on the location. Respondents in rural areas had a significantly higher proportion of being very worried about climate change compared to those in urban areas (19.9% vs. 15.6%). See Table 2 for details of the association between concern over climate change and gender and location.

**Table 2:** Climate change stratified by gender and location

**Experiences of Threats—Climate change**			**Total** ***N* = 2596**				**Total** ***N* = 2652**		
	**Category**		**Female** ***n* = 862**	**Male** ***n* = 1728**	***p*-value**^**d**^		**Rural** ***n* = 1627**	**Urban** ***n* = 1025**	***p*-value**^†^
I am worried that climate change threatens people and the planet	Not worried	598 (25.7%)	150 (19.3%)^a^	447 (29.0%)^b^	**< 0.001**	612 (25.9%)	371 (25.8%)^a^	241 (25.9%)^a^	**0.018**
	A little	520 (22.4%)	197 (25.3%)^a^	322 (20.9%)^b^		533 (22.5%)	306 (21.3%)^a^	227 (24.4%)^a^	
	Moderately	382 (16.4%)	116 (14.9%)^a^	265 (17.2%)^a^		383 (16.2%)	218 (15.2%)^a^	165 (17.7%)^a^	
	Very worried	425 (18.3%)	167 (21.5%)^a^	258 (16.7%)^b^		431 (18.2%)	286 (19.9%)^a^	145 (15.6%)^b^	
	Extremely worried	302 (13.0%)	104 (13.4%)^a^	198 (12.8%)^a^		307 (13.0%)	199 (13.9%)^a^	108 (11.6%)^a^	
	Prefer not to say	98 (4.2%)	44 (5.7%)^a^	54 (3.5%)^b^		100 (4.2%)	56 (3.9%)^a^	44 (4.7%)^a^	
Does climate change make you feel Anxious	Yes	814 (35.3%)	286 (38.2%)^a^	526 (33.9%)^b^	0.202	828 (35.4%)	495 (35.1%)^a^	333 (35.8%)^a^	0.908
	No	1,305 (56.6%)	407 (54.3%)^a^	897 (57.8%)^a^		1,326 (56.6%)	804 (57.0%)^a^	522 (56.1%)^a^	
	Prefer not to say	186 (8.1%)	56 (7.5%)^a^	130 (8.4%)^a^		188 (8.0%)	112 (7.9%)^a^	76 (8.2%)^a^	
Does climate change make you feel Angry	Yes	575 (25.4%)	204 (27.9%)^a^	370 (24.1%)^a^	0.217	588 (25.5%)	344 (24.7%)^a^	244 (26.7%)^a^	0.416
	No	1,533 (67.7%)	482 (66.0%)^a^	1,049 (68.4%)^a^		1,558 (67.6%)	954 (68.6%)^a^	604 (66.0%)^a^	
	Prefer not to say	158 (7.0%)	44 (6.0%)^a^	114 (7.4%)^a^		159 (6.9%)	92 (6.6%)^a^	67 (7.3%)^a^	
Does climate change make you feel Afraid	Yes	833 (36.5%)	313 (42.3%)^a^	520 (33.8%)^b^	**< 0.001**	846 (36.4%)	514 (36.5%)^a^	332 (36.4%)^a^	0.475
	No	1,267 (55.5%)	375 (50.7%)^a^	889 (57.7%)^b^		1,289 (55.5%)	774 (54.9%)^a^	515 (56.4%)^a^	
	Prefer not to say	183 (8.02%)	52 (7.0%)^a^	131 (8.51%)^a^		187 (8.1%)	121 (8.6%)^a^	66 (7.2%)^a^	
Does climate change make you feel Powerless	Yes	498 (22.0%)	181 (24.8%)^a^	317 (20.7%)^b^	0.209	507 (22.0%)	319 (22.9%)^a^	188 (20.6%)^a^	0.392
	No	1,508 (66.6%)	470 (64.3%)^a^	1,035 (67.7%)^a^		1,534 (66.6%)	913 (65.6%)^a^	621 (68.2%)^a^	
	Prefer not to say	257 (11.4%)	80 (10.9%)^a^	177 (11.6%)^a^		261 (11.3%)	159 (11.4%)^a^	102 (11.2%)^a^	

^‡^ = column percentage; † = chi-square test; d = Fisher’s exact test; *p*-value = significance level; Each subscript letter (a, b) denotes a subset of gender/location categories whose column proportions do not differ significantly from each other at the 0.05 level

### Climate change and SDQ emotional symptoms

Emotional symptoms on the SDQ were associated with all five concerns about climate change. All concerns about climate change (worry, anxiety, anger, fear, powerlessness) significantly increased the likelihood of severity of emotional symptoms. Being a little worried, very worried and extremely worried about climate change predicted an increase in emotional symptoms. See Table 3 for details of the association between concern over climate change and emotional difficulties.

**Table 3:** Climate change and SDQ Emotional symptoms

**Experiences of Threats—Climate change**	**Category**	**Total** ***N* = 2554**	**Emotional Symptoms**^‡^			***p*-value**^†^	**Univariate ordinal logistic regression**		**Multivariate ordinal logistic regression**	
			**average score 0–5: unlikely clinically significant problems** ***n* = 2218**	**slightly raised score 6: borderline clinically significant problems** ***n* = 136**	**high scores 7–10: substantial risk clinically significant problems** ***n* = 200**		**COR (95% CI)**	***p*-value**	**AOR (95% CI)**	***p*-value**
I am worried that climate change threatens people and the planet	Not worried	600 (26.0%)	560 (28.0%)^a^	17 (13.5%)^b^	23 (12.5%)^b^	**< 0.001**	Ref		Ref	
	A little	521 (22.5%)	453 (22.6%)^a^	28 (22.2%)^a^	40 (21.7%)^a^		2.03 (1.38–3.02)	**< 0.001**	1.73 (1.16–2.62)	**0.008**
	Moderately	374 (16.2%)	321 (16.0%)^a^	26 (20.6%)^a^	27 (14.7%)^a^		2.20 (1.46–3.34)	**< 0.001**	1.50 (0.95–2.36)	0.083
	Very worried	416 (18.0%)	351 (17.5%)^a^	21 (16.7%)^a,b^	44 (23.9%)^b^		2.48 (1.68–3.71)	**< 0.001**	1.73 (1.11–2.72)	**0.016**
	Extremely worried	300 (13.0%)	234 (11.7%)^a^	26 (20.6%)^b^	40 (21.7%)^b^		3.60 (2.44–5.38)	**< 0.001**	2.46 (1.58–3.87)	** < 0.001**
	Prefer not to say	100 (4.3%)	82 (4.1%)^a^	8 (6.4%)^a^	10 (5.4%)^a^		2.87 (1.60–4.92)	**< 0.001**	2.22 (1.20–3.96)	**0.009**
Does climate change make you feel Anxious	Yes	812 (35.2%)	666 (33.4%)^a^	56 (45.2%)^b^	90 (48.9%)^b^	**< 0.001**	1.82 (1.44–2.30)	**< 0.001**	1.18 (0.89–1.57)	0.255
	No	1,307 (56.7%)	1,172 (58.7%)^a^	58 (46.8%)^b^	77 (41.8%)^b^		Ref		Ref	
	Prefer not to say	185 (8.0%)	158 (7.9%)^a^	10 (8.1%)^a^	17 (9.2%)^a^		1.45 (0.94–2.16)	0.077	0.68 (0.39–1.16)	0.167
Does climate change make you feel Angry	Yes	577 (25.4%)	472 (24.0%)^a^	46 (37.1%)^b^	59 (33.1%)^b^	**< 0.001**	1.75 (1.36–2.23)	**< 0.001**	1.04 (0.77–1.40)	0.790
	No	1,533 (67.6%)	1,367 (69.5%)^a^	68 (54.8%)^b^	98 (55.1%)^b^		Ref		Ref	
	Prefer not to say	158 (7.0%)	127 (6.5%)^a^	10 (8.1%)^a,b^	21 (11.8%)^b^		1.92 (1.29–2.78)	**< 0.001**	1.34 (0.76–2.27)	0.297
Does climate change make you feel Afraid	Yes	835 (36.6%)	691 (34.9%)^a^	56 (45.9%)^b^	88 (48.4%)^b^	**< 0.001**	1.86 (1.46–2.36)	**< 0.001**	1.23 (0.92–1.66)	0.167
	No	1,267 (55.5%)	1,144 (57.8%)^a^	50 (41.0%)^b^	73 (40.1%)^b^		Ref		Ref	
	Prefer not to say	182 (8.0%)	145 (7.3%)^a^	16 (13.1%)^b^	21 (11.5%)^b^		2.22 (1.51–3.16)	**< 0.001**	1.36 (0.79–2.28)	0.248
Does climate change make you feel Powerless	Yes	499 (22.0%)	403 (20.5%)^a^	37 (30.6%)^b^	59 (32.6%)^b^	**< 0.001**	1.99 (1.54–2.56)	**< 0.001**	1.34 (0.98–1.84)	0.066
	No	1,510 (66.6%)	1,356 (69.0%)^a^	64 (52.9%)^b^	90 (49.7%)^b^		Ref		Ref	
	Prefer not to say	257 (11.3%)	205 (10.4%)^a^	20 (16.5%)^b^	32 (17.7%)^b^		2.10 (1.52–2.86)	**< 0.001**	1.50 (0.96–2.28)	0.064

*COR* Crude Odds Ratio, *AOR* Adjusted Odds Ratio, *CI* Confidence Interval, *Ref* Reference category

^‡^ = column percentage; † = chi-square test*; p*-value = significance level; Each subscript letter (a, b) denotes a subset of emotional symptoms categories whose column proportions do not differ significantly from each other at the 0.05 level

### Climate change and SDQ conduct problems

SDQ conduct problems were associated with four of the five concerns about climate change (worry, anxiety, anger, and powerlessness). Four emotions related to threats of climate change (worry, anxiety, anger, powerlessness) significantly increased the likelihood of severity in symptoms of conduct problems. On multivariate analyses, being very worried about climate change and being extremely worried about climate change were predictors of conduct problems. See Table 4 for details of the association between concerns over climate change and conduct problems.

**Table 4:** Climate change and SDQ Conduct problems

**Experiences of Threats—Climate change**	**Category**	**Total** ***N* = 2546**	**Conduct Problems**^‡^			***p*-value**^†^	**Univariate ordinal logistic regression**		**Multivariate ordinal logistic regression**	
			**average score 0–3: unlikely clinically significant problems** ***n* = 1773**	**slightly raised score 4: borderline clinically significant problems** ***n* = 372**	**high scores 5–10: substantial risk clinically significant problems** ***n* = 401**		**COR (95% CI)**	***p*-value**	**AOR (95% CI)**	***p*-value**
I am worried that climate change threatens people and the planet	Not worried	599 (25.9%)	446 (27.8%)^a^	80 (23.3%)^a,b^	73 (20.2%)^b^	**< 0.001**	Ref		Ref	
	A little	520 (22.5%)	383 (23.9%)^a^	71 (20.7%)^a,b^	66 (18.2%)^b^		1.04 (0.82–1.31)	0.757	0.99 (0.78–1.27)	0.954
	Moderately	375 (16.2%)	259 (16.1%)^a^	50 (14.6%)^a^	66 (18.2%)^a^		1.27 (1.00–1.62)	0.050	1.15 (0.88–1.50)	0.305
	Very worried	416 (18.0%)	263 (16.4%)^a^	69 (20.1%)^a,b^	84 (23.2%)^b^		1.57 (1.25–1.97)	**< 0.001**	1.41 (1.09–1.84)	**0.010**
	Extremely worried	300 (13.0%)	187 (11.7%)^a^	51 (14.9%)^a,b^	62 (17.1%)^b^		1.62 (1.27–2.06)	**< 0.001**	1.53 (1.15–2.02)	**0.003**
	Prefer not to say	100 (4.3%)	67 (4.2%)^a,b^	22 (6.4%)^b^	11 (3.0%)^a^		1.31 (0.89–1.89)	0.154	1.20 (0.79–1.77)	0.381
Does climate change make you feel Anxious	Yes	810 (35.2%)	536 (33.4%)^a^	128 (38.0%)^a,b^	146 (40.3%)^b^	**0.004**	1.30 (1.11–1.52)	**0.001**	1.12 (0.92–1.36)	0.263
	No	1,307 (56.8%)	950 (59.3%)^a^	179 (53.1%)^b^	178 (49.2%)^b^		Ref		Ref	
	Prefer not to say	185 (8.0%)	117 (7.3%)^a^	30 (8.9%)^a,b^	38 (10.5%)^b^		1.45 (1.11–1.87)	**0.005**	1.38 (0.98–1.93)	0.060
Does climate change make you feel Angry	Yes	576 (25.4%)	365 (23.2%)^a^	98 (29.3%)^b^	113 (31.9%)^b^	**< 0.001**	1.42 (1.20–1.67)	**< 0.001**	1.18 (0.96–1.45)	0.117
	No	1,531 (67.6%)	1,108 (70.3%)^a^	216 (64.5%)^b^	207 (58.5%)^b^		Ref		Ref	
	Prefer not to say	157 (6.9%)	102 (6.5%)^a^	21 (6.3%)^a,b^	34 (9.6%)^b^		1.37 (1.02–1.79)	**0.030**	0.93 (0.61–1.40)	0.729
Does climate change make you feel Afraid	Yes	835 (36.6%)	567 (35.8%)^a^	123 (36.7%)^a^	145 (39.9%)^a^	0.059	1.15 (0.98–1.35)	0.077	0.92 (0.76–1.13)	0.431
	No	1,265 (55.4%)	902 (56.9%)^a^	184 (54.9%)^a,b^	179 (49.3%)^b^		Ref		Ref	
	Prefer not to say	182 (8.0%)	115 (7.3%)^a^	28 (8.4%)^a,b^	39 (10.7%)^b^		1.38 (1.05–1.78)	**0.016**	1.08 (0.74–1.56)	0.678
Does climate change make you feel Powerless	Yes	499 (22.1%)	324 (20.6%)^a^	81 (24.3%)^a,b^	94 (26.3%)^b^	**0.001**	1.32 (1.10–1.57)	**0.002**	1.01 (0.80–1.26)	0.952
	No	1,507 (66.6%)	1,084 (69.0%)^a^	216 (64.7%)^a,b^	207 (58.0%)^b^		Ref		Ref	
	Prefer not to say	257 (11.4%)	164 (10.4%)^a^	37 (11.1%)^a,b^	56 (15.7%)^b^		1.39 (1.11–1.73)	**0.004**	1.13 (0.82–1.52)	0.447

*COR* Crude Odds Ratio, *AOR* Adjusted Odds Ratio, *CI* Confidence Interval, *Ref.* Reference category

^‡^ = column percentage; † = chi-square test*; p*-value = significance level; Each subscript letter (a, b) denotes a subset of conduct problem categories whose column proportions do not differ significantly from each other at the 0.05 level

### Climate change and hyperactivity

SDQ hyperactivity was associated with four of the five concerns about climate change (anxiety, anger, fear, and powerlessness). However, none of the climate change concerns predicted hyperactivity. See Table 5 for details of the association between concern over climate change and hyperactivity symptoms.

**Table 5:** Climate change and SDQ Hyperactivity

**Experiences of Threats—Climate change**	**Category**	**Total** ***N* = 2542**	**Hyperactivity**^‡^			***p*-value**	**Univariate ordinal logistic regression**		**Multivariate ordinal logistic regression**	
			**average score 0–5: unlikely clinically significant problems** ***n* = 2302**	**slightly raised score 6: borderline clinically significant problems** ***n* = 134**	**high scores 7–10: substantial risk clinically significant problems** ***n* = 106**		**COR (95% CI)**	***p*-value**	**AOR (95% CI)**	***p*-value**
I am worried that climate change threatens people and the planet	Not worried	597 (25.9%)	540 (25.9%)^a^	33 (26.0%)^a^	24 (24.7%)^a^	0.802^d^	Ref		Ref	
	A little	520 (22.5%)	480 (23.0%)^a^	19 (15.0%)^b^	21 (21.6%)^a,b^		0.80 (0.53–1.20)	0.283	0.81 (0.52–1.24)	0.332
	Moderately	375 (16.2%)	335 (16.1%)^a^	24 (18.9%)^a^	16 (16.5%)^a^		1.12 (0.74–1.68)	0.575	1.14 (0.73–1.78)	0.559
	Very worried	416 (18.0%)	373 (17.9%)^a^	25 (19.7%)^a^	18 (18.6%)^a^		1.09 (0.73–1.61)	0.680	1.05 (0.65–1.67)	0.839
	Extremely worried	300 (13.0%)	267 (12.8%)^a^	20 (15.7%)^a^	13 (13.4%)^a^		1.16 (0.75–1.77)	0.499	1.13 (0.68–1.85)	0.620
	Prefer not to say	100 (4.3%)	89 (4.3%)^a^	6 (4.7%)^a^	5 (5.2%)^a^		1.16 (0.58–2.13)	0.646	0.96 (0.45–1.87)	0.910
Does climate change make you feel Anxious	Yes	813 (35.3%)	729 (35.1%)^a^	51 (41.5%)^a^	33 (33.7%)^a^	**0.047**^†^	1.23 (0.93–1.63)	0.150	1.11 (0.78–1.56)	0.570
	No	1,304 (56.7%)	1,194 (57.4%)^a^	57 (46.3%)^b^	53 (54.1%)^a,b^		Ref		Ref	
	Prefer not to say	183 (8.0%)	156 (7.5%)^a^	15 (12.2%)^a^	12 (12.2%)^a^		1.81 (1.16–2.71)	**0.006**	1.15 (0.63–2.02)	0.647
Does climate change make you feel Angry	Yes	577 (25.5%)	517 (25.3%)^a^	29 (23.6%)^a^	31 (31.6%)^a^	**0.034**^†^	1.19 (0.87–1.60)	0.272	1.15 (0.80–1.66)	0.445
	No	1,530 (67.6%)	1,394 (68.3%)^a^	80 (65.0%)^a,b^	56 (57.1%)^b^		Ref		Ref	
	Prefer not to say	156 (6.9%)	131 (6.4%)^a^	14 (11.4%)^b^	11 (11.2%)^a,b^		1.88 (1.20–2.82)	**0.004**	1.24 (0.63–2.35)	0.529
Does climate change make you feel Afraid	Yes	835 (36.6%)	757 (36.8%)^a^	50 (40.3%)^a^	28 (28.6%)^a^	**0.029**^†^	1.01 (0.76–1.34)	0.940	0.86 (0.60–1.23)	0.405
	No	1,263 (55.4%)	1,147 (55.7%)^a^	58 (46.8%)^a^	58 (59.2%)^a^		Ref		Ref	
	Prefer not to say	182 (8.0%)	154 (7.5%)^a^	16 (12.9%)^b^	12 (12.2%)^a,b^		1.73 (1.12–2.57)	**0.010**	1.01 (0.53–1.87)	0.967
Does climate change make you feel Powerless	Yes	498 (22.0%)	452 (22.1%)^a^	24 (20.0%)^a^	22 (22.4%)^a^	**0.006**^†^	1.07 (0.76–1.48)	0.697	1.01 (0.67–1.51)	0.953
	No	1,508 (66.7%)	1,377 (67.4%)^a^	75 (62.5%)^a,b^	56 (57.1%)^b^		Ref		Ref	
	Prefer not to say	256 (11.3%)	215 (10.5%)^a^	21 (17.5%)^b^	20 (20.4%)^b^		1.93 (1.34–2.71)	** < 0.001**	1.60 (0.96–2.56)	0.060

*COR* Crude Odds Ratio, *AOR* Adjusted Odds Ratio, *CI* Confidence Interval, *Ref.* Reference category

^‡^ = column percentage; † = chi-square test*;* d = Fisher’s exact test; *p*-value = significance level; Each subscript letter (a, b) denotes a subset of hyperactivity categories whose column proportions do not differ significantly from each other at the 0.05 level

### Climate change and peer problems

SDQ peer problems scores were associated with four of the five concerns about climate change (worry, anxiety, anger, powerlessness). Three concerns of climate change (worry, anxiety, anger) significantly increased the likelihood of severity of peer problems. Being extremely worried about climate change was a predictor of peer problems. See Table 6 for details of the association between concern over climate change and peer problems.

**Table 6:** Climate change and Peer problems

**Experiences of Threats—Climate change**	**Category**	**Total** ***N* = 2541**	**Peer Problems**^‡^			***p*-value**^**†**^	**Univariate ordinal logistic regression**		**Multivariate ordinal logistic regression**	
			**average score 0–3: unlikely clinically significant problems** ***n* = 1750**	**slightly raised score 4–5: borderline clinically significant problems** ***n* = 559**	**high scores 6–10: substantial risk clinically significant problems** ***n* = 232**		**COR (95% CI)**	***p*-value**	**AOR (95% CI)**	***p*-value**
I am worried that climate change threatens people and the planet	Not worried	596 (25.9%)	437 (27.5%)^a^	119 (23.5%)^a,b^	40 (19.1%)^b^	**0.024**	Ref		Ref	
	A little	517 (22.4%)	362 (22.8%)^a^	108 (21.3%)^a^	47 (22.5%)^a^		1.16 (0.93–1.44)	0.197	1.13 (0.90–1.44)	0.295
	Moderately	373 (16.2%)	265 (16.7%)^a^	74 (14.6%)^a^	34 (16.3%)^a^		1.11 (0.87–1.42)	0.395	0.99 (0.75–1.30)	0.935
	Very worried	417 (18.1%)	278 (17.5%)^a^	94 (18.5%)^a^	45 (21.5%)^a^		1.32 (1.05–1.66)	**0.017**	1.24 (0.95–1.62)	0.107
	Extremely worried	301 (13.1%)	184 (11.6%)^a^	84 (16.6%)^b^	33 (15.8%)^a,b^		1.59 (1.25–2.01)	** < 0.001**	1.44 (1.09–1.91)	**0.010**
	Prefer not to say	100 (4.3%)	62 (3.9%)^a^	28 (5.5%)^a^	10 (4.8%)^a^		1.53 (1.06–2.16)	**0.018**	1.56 (1.06–2.25)	**0.020**
Does climate change make you feel Anxious	Yes	810 (35.2%)	535 (33.8%)^a^	206 (40.6%)^b^	69 (33.2%)^a,b^	**0.043**	1.19 (1.02–1.39)	**0.027**	1.06 (0.87–1.29)	0.545
	No	1,304 (56.7%)	925 (58.4%)^a^	261 (51.5%)^b^	118 (56.7%)^a,b^		Ref		Ref	
	Prefer not to say	185 (8.1%)	124 (7.8%)^a^	40 (7.9%)^a^	21 (10.1%)^a^		1.17 (0.89–1.52)	0.251	0.91 (0.63–1.29)	0.610
Does climate change make you feel Angry	Yes	575 (25.4%)	372 (23.9%)^a^	142 (28.6%)^b^	61 (29.5%)^a,b^	**0.048**	1.27 (1.07–1.49)	**0.006**	1.07 (0.87–1.31)	0.548
	No	1,530 (67.6%)	1,084 (69.6%)^a^	319 (64.2%)^b^	127 (61.4%)^b^		Ref		Ref	
	Prefer not to say	157 (6.9%)	102 (6.6%)^a^	36 (7.2%)^a^	19 (9.2%)^a^		1.26 (0.94–1.66)	0.101	0.96 (0.63–1.44)	0.845
Does climate change make you feel Afraid	Yes	834 (36.6%)	562 (35.9%)^a^	195 (39.1%)^a^	77 (36.5%)^a^	0.106	1.14 (0.97–1.33)	0.113	1.02 (0.84–1.24)	0.836
	No	1,261 (55.4%)	892 (56.9%)^a^	258 (51.7%)^b^	111 (52.6%)^a,b^		Ref		Ref	
	Prefer not to say	182 (8.0%)	113 (7.2%)^a^	46 (9.2%)^a^	23 (10.9%)^a^		1.38 (1.06–1.78)	**0.014**	1.25 (0.85–1.80)	0.241
Does climate change make you feel Powerless	Yes	498 (22.0%)	329 (21.2%)^a^	121 (24.4%)^a^	48 (22.7%)^a^	**0.034**	1.18 (0.99–1.41)	0.064	1.00 (0.80–1.24)	0.978
	No	1,505 (66.6%)	1,061 (68.3%)^a^	316 (63.8%)^a,b^	128 (60.7%)^b^		Ref		Ref	
	Prefer not to say	256 (11.3%)	163 (10.5%)^a^	58 (11.7%)^a,b^	35 (16.6%)^b^		1.31 (1.04–1.63)	**0.017**	1.12 (0.81–1.53)	0.471

*COR* Crude Odds Ratio, *AOR* Adjusted Odds Ratio, *CI* Confidence Interval, *Ref.* Reference category

^‡^ = column percentage; † = chi-square test*; p*-value = significance level; Each subscript letter (a, b) denotes a subset of peer problem categories whose column proportions do not differ significantly from each other at the 0.05 level

### Climate change and prosocial behavior

SDQ prosocial behavior scores were associated with three of the five concerns about climate change (worry, anger, fear). Three threats of climate change (worry, anger, fear) significantly reduced the likelihood of high scores on prosocial behavior. Being afraid of climate change was a predictor of reduced prosocial behavior. See Table 7 for details of the association between concern about climate change and prosocial behavior.

**Table 7:** Climate change and SDQ Prosocial behavior

**Experiences of Threats—Climate change**	**Category**	**Total** ***N* = 2554**	**Prosocial Behavior**^‡^			***p*-value**^**†**^	**Univariate ordinal logistic regression**		**Multivariate ordinal logistic regression**	
			**average score 6–10: unlikely clinically significant problems** ***n* = 2039**	**slightly low score 5: borderline clinically significant problems** ***n* = 240**	**low scores 0–4: substantial risk clinically significant problems** ***n* = 275**		**COR (95% CI)**	***p*-value**	**AOR (95% CI)**	***p*-value**
I am worried that climate change threatens people and the planet	Not worried	600 (25.9%)	455 (24.4%)^a^	64 (29.6%)^a,b^	81 (34.8%)^b^	**0.031**	Ref		Ref	
	A little	521 (22.5%)	429 (23.0%)^a^	50 (23.1%)^a^	42 (18.0%)^a^		0.70 (0.53–0.90)	**0.006**	0.80 (0.61–1.06)	0.122
	Moderately	375 (16.2%)	303 (16.3%)^a^	39 (18.1%)^a^	33 (14.2%)^a^		0.76 (0.57–1.01)	0.060	0.91 (0.66–1.23)	0.531
	Very worried	417 (18.0%)	339 (18.2%)^a^	31 (14.4%)^a^	47 (20.2%)^a^		0.75 (0.57–0.99)	**0.042**	0.96 (0.70–1.32)	0.814
	Extremely worried	300 (13.0%)	255 (13.7%)^a^	23 (10.6%)^a^	22 (9.4%)^a^		0.58 (0.41–0.81)	**0.002**	0.69 (0.46–1.00)	0.057
	Prefer not to say	100 (4.3%)	83 (4.5%)^a^	9 (4.2%)^a^	8 (3.4%)^a^		0.67 (0.39–1.07)	0.115	0.88 (0.50–1.43)	0.617
Does climate change make you feel Anxious	Yes	814 (35.3%)	665 (35.9%)^a^	76 (35.2%)^a^	73 (30.4%)^a^	0.090	0.83 (0.68–1.02)	0.073	0.99 (0.77–1.26)	0.924
	No	1,307 (56.7%)	1,027 (55.5%)^a^	126 (58.3%)^a,b^	154 (64.2%)^b^		Ref		Ref	
	Prefer not to say	185 (8.0%)	158 (8.5%)^a^	14 (6.5%)^a^	13 (5.4%)^a^		0.65 (0.43–0.95)	**0.033**	0.71 (0.42–1.14)	0.172
Does climate change make you feel Angry	Yes	575 (25.4%)	481 (26.6%)^a^	47 (21.9%)^a,b^	47 (19.6%)^b^	**0.027**	0.72 (0.57–0.90)	**0.005**	0.78 (0.59–1.02)	0.073
	No	1,534 (67.7%)	1,197 (66.1%)^a^	157 (73.0%)^b^	180 (75.0%)^b^		Ref		Ref	
	Prefer not to say	157 (6.9%)	133 (7.3%)^a^	11 (5.1%)^a^	13 (5.4%)^a^		0.67 (0.43–0.99)	0.058	0.56 (0.31–0.99)	0.052
Does climate change make you feel Afraid	Yes	837 (36.6%)	708 (38.7%)^a^	64 (29.9%)^b^	65 (27.0%)^b^	**0.001**	0.65 (0.53–0.80)	** < 0.001**	0.76 (0.59–0.99)	**0.040**
	No	1,267 (55.4%)	980 (53.6%)^a^	134 (62.6%)^b^	153 (63.5%)^b^		Ref		Ref	
	Prefer not to say	181 (7.9%)	142 (7.8%)^a^	16 (7.5%)^a^	23 (9.5%)^a^		0.95 (0.67–1.31)	0.771	1.18 (0.73–1.86)	0.483
Does climate change make you feel Powerless	Yes	500 (22.1%)	413 (22.8%)^a^	44 (20.4%)^a^	43 (17.8%)^a^	0.363	0.82 (0.64–1.03)	0.102	1.15 (0.85–1.53)	0.352
	No	1,509 (66.6%)	1,196 (66.1%)^a^	148 (68.5%)^a^	165 (68.5%)^a^		Ref		Ref	
	Prefer not to say	257 (11.3%)	200 (11.1%)^a^	24 (11.1%)^a^	33 (13.7%)^a^		1.09 (0.81–1.43)	0.569	1.54 (1.04–2.22)	**0.027**

*COR* Crude Odds Ratio, *AOR* Adjusted Odds Ratio, *CI* Confidence Interval, *Ref*. Reference category

^‡^ = column percentage; † = chi-square test*; p*-value = significance level; Each subscript letter (a, b) denotes a subset of prosocial behavior categories whose column proportions do not differ significantly from each other at the 0.05 level

### Climate change and SDQ total difficulties

SDQ total difficulties were associated with all five concerns about climate change. All threats of climate change (worry, anxiety, anger, fear, powerlessness) significantly increased the likelihood on severity in symptoms of mental difficulties. Two of the threats of climate change – worry and anger predicted total mental difficulties. See Table 8 for details of the association between climate change concerns and total mental difficulties.

**Table 8:** Climate change and SDQ Total difficulties

**Experiences of Threats—Climate change**	**Category**	**Total** ***N* = 2543**	**Total Difficulties**^‡^			***p*-value**^**†**^	**Univariate ordinal logistic regression**		**Multivariate ordinal logistic regression**	
			**average score 0–15: unlikely clinically significant problems** ***n* = 2024**	**slightly raised score 16–19: borderline clinically significant problems** ***n* = 299**	**high scores 20–40: substantial risk clinically significant problems** ***n* = 220**		**COR (95% CI)**	***p*-value**	**AOR (95% CI)**	***p*-value**
I am worried that climate change threatens people and the planet	Not worried	597 (25.9%)	509 (27.8%)^a^	54 (19.6%)^b^	34 (16.9%)^b^	**< 0.001**	Ref		Ref	
	A little	518 (22.5%)	422 (23.1%)^a^	59 (21.4%)^a^	37 (18.4%)^a^		1.28 (0.96–1.72)	0.091	1.21 (0.89–1.65)	0.219
	Moderately	374 (16.2%)	295 (16.1%)^a^	45 (16.3%)^a^	34 (16.9%)^a^		1.49 (1.10–2.02)	**0.010**	1.22 (0.87–1.72)	0.245
	Very worried	417 (18.1%)	310 (16.9%)^a^	61 (22.1%)^a^	46 (22.9%)^b^		1.86 (1.41–2.48)	** < 0.001**	1.51 (1.09–2.11)	**0.014**
	Extremely worried	300 (13.0%)	214 (11.7%)^a^	46 (16.7%)^b^	40 (19.9%)^b^		2.13 (1.58–2.87)	** < 0.001**	1.74 (1.23–2.46)	**0.002**
	Prefer not to say	100 (4.3%)	79 (4.3%)^a^	11 (4.0%)^a^	10 (5.0%)^a^		1.49 (0.90–2.35)	0.100	1.26 (0.74–2.06)	0.370
Does climate change make you feel Anxious	Yes	810 (35.2%)	609 (33.3%)^a^	112 (41.8%)^b^	89 (43.8%)^b^	**< 0.001**	1.49 (1.23–1.80)	** < 0.001**	1.10 (0.87–1.39)	0.420
	No	1,305 (56.7%)	1,077 (58.9%)^a^	141 (52.6%)^a^	87 (42.9%)^b^		Ref		Ref	
	Prefer not to say	185 (8.0%)	143 (7.8%)^a^	15 (5.6%)^a^	27 (13.3%)^b^		1.38 (0.98–1.90)	0.053	0.75 (0.48–1.15)	0.203
Does climate change make you feel Angry	Yes	576 (25.5%)	416 (23.2%)^a^	96 (35.4%)^b^	64 (32.7%)^b^	**< 0.001**	1.74 (1.43–2.12)	** < 0.001**	1.29 (1.01–1.65)	**0.040**
	No	1,530 (67.6%)	1,270 (70.7%)^a^	153 (56.5%)^b^	107 (54.6%)^b^		Ref		Ref	
	Prefer not to say	157 (6.9%)	110 (6.1%)^a^	22 (8.1%)^a,b^	25 (12.8%)^b^		1.94 (1.41–2.63)	** < 0.001**	1.33 (0.83–2.09)	0.234
Does climate change make you feel Afraid	Yes	834 (36.6%)	647 (35.8%)^a^	106 (39.3%)^a^	81 (40.5%)^a^	**< 0.001**	1.28 (1.06–1.56)	**0.012**	0.90 (0.70–1.14)	0.377
	No	1,263 (55.4%)	1,036 (57.3%)^a^	134 (49.6%)^b^	93 (46.5%)^b^		Ref		Ref	
	Prefer not to say	182 (8.0%)	126 (7.0%)^a^	30 (11.1%)^b^	26 (13.0%)^b^		1.87 (1.38–2.48)	** < 0.001**	1.15 (0.73–1.76)	0.530
Does climate change make you feel Powerless	Yes	499 (22.1%)	367 (20.5%)^a^	84 (31.1%)^b^	48 (24.2%)^a,b^	**< 0.001**	1.64 (1.33–2.02)	** < 0.001**	1.24 (0.95–1.61)	0.114
	No	1,506 (66.6%)	1,251 (69.7%)^a^	149 (55.2%)^b^	106 (53.5%)^b^		Ref		Ref	
	Prefer not to say	257 (11.4%)	176 (9.8%)^a^	37 (13.7%)^b^	44 (22.2%)^c^		2.08 (1.61–2.66)	** < 0.001**	1.68 (1.17–2.36)	**0.004**

*COR* Crude Odds Ratio, *AOR* Adjusted Odds Ratio, *CI* Confidence Interval, *Ref.* Reference category

^‡^ = column percentage; † = chi-square test*; p*-value = significance level; Each subscript letter (a, b, c) denotes a subset of total difficulties categories whose column proportions do not differ significantly from each other at the 0.05 level

### Suicidality and threats of climate change

Suicidal thoughts were associated with all five concerns about climate change. On multivariate analyses being worried about climate change and feeling powerless about climate change were predictors of suicidal thoughts.

Suicide plans were associated with all five concerns about climate change. Being extremely worried about climate change predicted suicide plans.

Four of the five concerns about climate change were significantly associated with attempted suicide while three of the concerns of climate change – worry, anxiety and powerlessness predicted suicide attempt. See Table 9 for associations between climate change concerns and suicidality.

**Table 9:** Suicidality and Threats of climate change

Experiences of Threats—Climate change	Category	Overall (*N* = 2652)	Total (*N* = 2534)	Suicide Thought^†^	Suicide Plan^†^	Suicide Attempt^†^	Univariate logistic regression			Multivariate logistic regression		
				**(*n* = 680)**	**(*n* = 377)**	**(*n* = 397)**	**Suicide Thought**	**Suicide Plan**	**Suicide Attempt**	**Suicide Thought**	**Suicide Plan**	**Suicide Attempt**
							**COR (95% CI)**	**COR (95% CI)**	**COR (95% CI)**	**AOR (95% CI)**	**AOR (95% CI)**	**AOR (95% CI)**
I am worried that climate change threatens people and the planet	Not worried	612 (25.9%)	602 (25.9%)	119 (19.8%)	67 (11.1%)	74 (12.3%)	Ref	Ref	Ref	Ref	Ref	Ref
	A little	533 (22.5%)	525 (22.6%)	137 (26.1%)	74 (14.1%)	71 (13.5%)	1.43 (1.08–1.90)*	1.31 (0.92–1.87)	1.12 (0.79–1.58)	1.40 (1.04–1.89)*	1.29 (0.88–1.89)	1.10 (0.76–1.59)
	Moderately	383 (16.2%)	375 (16.1%)	114 (30.4%)	63 (16.8%)	60 (16%)	1.77 (1.32–2.39)***	1.61 (1.11–2.34)*	1.36 (0.94–1.96)	1.55 (1.11–2.16)**	1.36 (0.89–2.06)	1.23 (0.82–1.84)
	Very worried	431 (18.2%)	421 (18.1%)	115 (27.3%)	64 (15.2%)	72 (17.1%)	1.53 (1.14–2.05)**	1.43 (0.99–2.07)	1.47 (1.04–2.09)*	1.24 (0.88–1.75)	1.16 (0.75–1.79)	1.28 (0.85–1.93)
	Extremely worried	307 (13.0%)	305 (13.1%)	109 (35.7%)	66 (21.6%)	62 (20.3%)	2.26 (1.66–3.07)***	2.21 (1.52–3.20)***	1.82 (1.26–2.63)**	2.01 (1.40–2.87)***	1.97 (1.27–3.05)**	1.71 (1.11–2.61)*
	Prefer not to say	100 (4.2%)	98 (4.2%)	24 (24.5%)	13 (13.3%)	16 (16.3%)	1.32 (0.78–2.15)	1.22 (0.62–2.24)	1.39 (0.75–2.45)	1.19 (0.68–2.02)	1.20 (0.58–2.32)	1.36 (0.70–2.51)
Does climate change make you feel Anxious	Yes	828 (35.4%)	812 (35.2%)	250 (30.8%)	140 (17.2%)	146 (18%)	1.35 (1.11–1.64)**	1.28 (1.00–1.62)*	1.28 (1.01–1.62)*	1.03 (0.81–1.30)	0.95 (0.71–1.28)	1.01 (0.76–1.35)
	No	1326 (56.6%)	1306 (56.7%)	323 (24.7%)	183 (14%)	191 (14.6%)	Ref	Ref	Ref	Ref	Ref	Ref
	Prefer not to say	188 (8.0%)	186 (8.1%)	49 (26.3%)	23 (12.4%)	23 (12.4%)	1.09 (0.76–1.53)	0.87 (0.53–1.35)	0.82 (0.51–1.28)	0.65 (0.41–1.03)	0.50 (0.26–0.90)*	0.50 (0.27–0.90)*
Does climate change make you feel Angry	Yes	588 (25.5%)	580 (25.6%)	187 (32.2%)	111 (19.1%)	104 (17.9%)	1.49 (1.21–1.84)***	1.57 (1.22–2.03)***	1.30 (1.00–1.67)*	1.16 (0.90–1.50)	1.31 (0.95–1.78)	1.02 (0.74–1.39)
	No	1558 (67.6%)	1529 (67.5%)	370 (24.2%)	200 (13.1%)	220 (14.4%)	Ref	Ref	Ref	Ref	Ref	Ref
	Prefer not to say	159 (6.9%)	157 (6.9%)	53 (33.8%)	27 (17.2%)	30 (19.1%)	1.60 (1.12–2.26)**	1.38 (0.87–2.11)	1.41 (0.91–2.12)	1.38 (0.83–2.28)	1.54 (0.83–2.82)	1.48 (0.81–2.65)
Does climate change make you feel Afraid	Yes	846 (36.4%)	835 (36.6%)	253 (30.3%)	148 (17.7%)	141 (16.9%)	1.34 (1.10–1.63)**	1.46 (1.14–1.85)**	1.18 (0.93–1.49)	1.07 (0.83–1.36)	1.25 (0.92–1.70)	0.94 (0.69–1.26)
	No	1289 (55.5%)	1264 (55.4%)	310 (24.5%)	163 (12.9%)	186 (14.7%)	Ref	Ref	Ref	Ref	Ref	Ref
	Prefer not to say	187 (8.1%)	184 (8.1%)	54 (29.3%)	31 (16.8%)	31 (16.8%)	1.28 (0.90–1.79)	1.37 (0.89–2.06)	1.17 (0.76–1.76)	0.84 (0.51–1.35)	1.17 (0.63–2.08)	0.80 (0.44–1.43)
Does climate change make you feel Powerless	Yes	507 (22.0%)	500 (22.1%)	160 (32%)	90 (18%)	98 (19.6%)	1.48 (1.19–1.85)***	1.37 (1.04–1.79)*	1.54 (1.18–2.00)**	1.19 (0.90–1.56)	0.96 (0.68–1.35)	1.38 (0.99–1.92)
	No	1534 (66.6%)	1506 (66.5%)	363 (24.1%)	208 (13.8%)	206 (13.7%)	Ref	Ref	Ref	Ref	Ref	
	Prefer not to say	261 (11.3%)	259 (11.4%)	94 (36.3%)	46 (17.8%)	55 (21.2%)	1.79 (1.35–2.37)***	1.35 (0.94–1.90)	1.70 (1.21–2.36)**	1.76 (1.21–2.56)**	1.12 (0.69–1.78)	1.71 (1.08–2.65)*

*COR* Crude Odds Ratio, *AOR* Adjusted Odds Ratio, *CI* Confidence Interval, *Ref*. Reference category

^*^p < 0.05

^**^p < 0.01

^***^p < 0.001

^†^row percentage

## Discussion

Our study was more on the association between climate change, mental disorders, and suicidality association rather than socio-economic predictors of the different associations. This to our knowledge is among the first Kenyan study to provide primary data on how climate change may be associated with mental health challenges of high school children. Our discussion in the Kenyan context is thus limited by the unavailability of previous Kenyan data for comparison.

### The response rate

The high response rate of 97.9% (*n* = 2596 out of 2652 participants) is not unique to this study. It has been consistently found in Kenyan studies and in particular in school-going children [31, 32]. There are several explanations: our approach to explain the nature of the study to the schools and communities, and the willingness to participate in any activity that has the potential to improve mental health and more specifically the mental health of students and in turn hopefully improving their academic performance.

### Social demographics

The gender disparity of 66.6% male and 33.2% female is a reflection of the schools recruited –more all-boys schools. It can also be a reflection of overall gender access to school in Kenya- girls are less likely to transition to secondary school and more likely to drop out due to factors such as early marriage, pregnancy, poverty, and cultural norms [33]. The decreasing number with years in high school could be a reflection of drop out over time or the availability of Form 4 students (the final year students) who may have opted not to participate in the study due to preparation for their final year exam. The 61% of students from rural areas, as opposed to 38.7% from urban areas, is a reflection of our deliberate effort to reach out to rural schools, the most vulnerable to the effects of climate change.

### Threats of climate change

The high response rate to the five questions on climate change can be explained in two ways. First, the response rate in previous school based studies is similar to what was found in our study [31, 32]. Secondly, the students were aware of the ongoing local and global concerns of climate change due to direct exposure, media exposure or word of mouth. More specifically, the students saw the effects of climate change on their lives i.e. loss of livelihoods in their families and communities in which they live due to prolonged droughts leading to loss of crops, death of livestock and decreased availability of water and food. Nearly 70% of the students agreed that “they are worried that climate change threatens people and the planet” that is they had a prospective perception of climate change and the need to do something to avert the threat. It is noteworthy that 22–36% of the students also had varying levels of immediate subjective emotional response to climate change.

### Gender and location

Females tend to have higher rates of internalizing symptoms [34] and this may explain why females were more worried (very worried to extremely worried) and afraid about climate change than males, a finding similar to that of a previous study [18]. There may also be gendered emotion norms at play here: it may be more accepted for females in societies to express worry and sadness [35]. It is not surprising that students at rural schools on average felt the threats of climate change because rural areas are more subject to immediate and highly visible effects of climate change for example death of livestock due to lack of fodder and water shortages and reduced crop production and the resulting economic difficulties and disruption of normal life.

### Climate change and SDQ scores

It is noteworthy that the worse the experiences of climate change, the higher were the scores on SDQ emotional symptoms, suggesting a direct positive relationship between the severity of climate change experiences and emotional symptoms. This was confirmed by univariate ordinal logistic regression for most of the associations studied. Indeed, being worried about the “threat of climate change to people and the planet” was the most significant predictor. This trend was repeated but to a lesser degree with conduct problems, hyperactivity, prosocial behavior and peer problems suggesting that climate change may have exacerbated these SDQ scores. There could also be links with the issue of meaning. Young people may think “why go with the status quo and societal/educational institution rules if the world is going to burn?” hence the reason why being very worried about climate change and being extremely worried about climate change were predictors of conduct problems.

Overall, experience of threats of climate change was associated with a significant increase in total SDQ difficulties. Our findings therefore, suggest climate change has a significant impact on the mental health of the adolescents that we studied. This concurs with findings of studies from other countries as documented in the introduction [6, 7, 9, 11–15]. Significantly, these high scores on SDQ difficulties were positively associated with suicidal thoughts, plans and attempts and also predicted suicidality.

The findings of this study suggest that climate change has mental health consequences and these consequences may lead to suicidality in Kenyan high school students, findings similar to previous studies that found a link between climate change and suicidality [4, 5]. These findings give impetus to the concerns of climate change and the need to reverse the trend for mental health reasons in Kenya. Future qualitative and quantitative studies may enrich our understanding of the mechanistic pathways to mental illness. This may be a fruitful area for research including biomarkers together with psychological assessments to inform the development of models to explain how youths respond to perceived and actual climate change.

A major limitation was that this was a cross-sectional study meaning no causalities were studied. Further, no diagnostic interviews were done and information was gathered only from the adolescents themselves and not parents. The study involved bias selection towards rural areas with a disadvantage of gender disparity in favor of boys but with the advantage that rural areas are the most affected by climate change such as loss of agricultural and livestock subsistence activities and therefore reduced income and availability of food.

## Data Availability

Requests for the data may be sent to the corresponding author.
